# Sources of soapy off‐flavor in clear acidic whey protein beverages

**DOI:** 10.1111/1750-3841.70044

**Published:** 2025-03-06

**Authors:** Anita R. Best, Yaozheng Liu, Dylan Cadwallader, MaryAnne Drake

**Affiliations:** ^1^ Southeast Dairy Foods Research Center, Department of Food, Bioprocessing, and Nutrition Sciences North Carolina State University Raleigh North Carolina USA

**Keywords:** clear acidic whey protein beverages, gas chromatography mass spectrometry, gas chromatography olfactometry, off‐flavor, solvent‐assisted flavor evaporation

## Abstract

**Abstract:**

Clear acidic protein beverages are made with whey protein isolate (WPI) due to its solubility at pH 3.3. These beverages can display a soapy off‐flavor, which is not detected at neutral pH. Our hypothesis was that medium chain fatty acids are the source of this off‐flavor. WPI (14 duplicate lots) were rehydrated to 10% solids (w/v), subjected to descriptive sensory analysis, and evaluated at their original pH, as well as pH adjusted to 6.3 (pre‐acidif) or to 3.3 (neutral) at 21°C. Solvent‐assisted flavor evaporation with gas chromatography mass spectrometry and gas chromatography olfactometry were performed on six selected WPIs (three neutral and three pre‐acidified), followed by quantitation of octanoic, decanoic, and dodecanoic acids using stable isotope dilution assays. Retronasal and orthonasal thresholds of these acids were determined at pH 6.3 and 3.3. Model systems of WPI with the three acids were generated to confirm soapy flavor. The average concentrations of the three acids in selected WPIs were as follows: C8 = 14.4 ± 2.0 ppm; C10 = 27.8 ± 2.39 ppm; C12 = 24.7 ± 2.20 ppm. Sensory detection thresholds for all three acids at pH 3.3 were lower than at pH 6.3 (*p* < 0.05). Trained panel profiling of model systems demonstrated that a combination of octanoic and decanoic acids within the concentration range documented in WPI at pH 3.3 caused a soapy flavor, which was not detected at pH 6.3. Identification of the sources of soapy off‐flavor in clear, acidic protein beverages provides the baseline to reduce or eliminate this off‐flavor.

**Practical Application:**

Clear acidic protein beverages are one category of protein beverages within the expanding world of protein beverages. Identification of the source of soapy off‐flavor in clear, acidic whey protein beverages is useful for consistent delivery of protein beverages that are desirable to consumers.

## 1 INTRODUCTION

The global sports food and drinks market was valued at $79.8 billion in 2020 and is expected to grow to $115.2 billion by the year 2026 (Businesswire, 2022). Protein beverages, a portion of this growing market, consist of both ready‐to‐drink (RTD) and ready‐to‐mix (RTM) products. Protein beverages consist of both ready‐to‐drink (RTD) and ready‐to‐mix (RTM) products. RTM beverages are made with instantized protein powders and can be mixed with water or another liquid of choice (B. J. Wright et al., 2009; Zhang et al., 2020). The RTD beverage category encompasses both neutral pH and acidified beverages (Carter et al., 2020; Singh et al., 2022). Neutral pH protein beverages fall between a pH of 6.6 and 7.0, while acidified protein beverages are lowered to a pH of <4.6, typically <3.5 (Carter et al., 2020). Neutral pH protein beverages encompass a wide array of dairy and plant protein sources. In contrast, acidified RTD beverages are limited to whey protein due to its unique solubility and clarity under acidic conditions. These clear acidic protein RTD beverages are typically flavored with fruity flavors and can be shelf‐stable with a mild hot fill heat treatment due to their low pH (Singh et al., 2022). Whey protein isolate (WPI) is the whey protein ingredient typically used in these beverages, and regular neutral pH WPI can be used, followed by addition with citric or phosphoric acids or a pre‐acidified spray‐dried WPI can be used. Commercial pre‐acidified WPI consist of WPI that has been acidified with citric and/or phosphoric acids prior to spray drying by the manufacturer.

As with other consumer products, flavor is a paramount attribute in RTD beverages, and protein‐related flavors in these beverages are a challenge (Harwood & Drake, 2019; Oltman et al., 2015; Liu et al., 2021). Both plant and dairy proteins, including whey protein, can contribute undesirable flavors to ingredient applications (Carter et al., 2020; Liu et al., 2023). Identifying sources of off‐flavors in RTD beverages or their protein sources can be useful to identify methods to reduce off‐flavors. For example, cardboard flavor is a ubiquitous flavor in dairy proteins—and proteins in general—and has been sourced to lipid oxidation products (Russell et al., 2006; Carter & Drake, 2018; Liu et al., 2023; Whitson et al., 2010). Minimizing process steps or storage to reduce oxidation can reduce the intensity of this off‐flavor in dried protein ingredients (Carter & Drake, 2018). Sources of cabbage and sulfur/eggy flavor in dairy proteins have been similarly identified from sulfur‐containing amino acids (Whitt et al., 2022; J. M. Wright et al., 2006). Soapiness is an off‐flavor in whey protein that has been attributed to solutions of whey protein (Oltman et al., 2015; White et al., 2013), but more prevalently in clear acidic protein beverages made with whey protein (Evans et al., 2010; Park et al., 2014).

Carunchia Whetstine et al. (2005) conducted one of the first studies to document the sensory profiles and flavor chemistry of WPI and whey protein concentrates (WPC80). Soapy flavor was only documented in two of the WPI evaluated. These WPIs had a higher pH than other WPI and a distinct mineral content. Carunchia Whetstine et al. (2005) identified octanoic and decanoic acids as flavor‐active in these WPI and suggested they might contribute to soapy/waxy flavor. White et al. (2013) demonstrated that acidified WPI (pH 3.2) had increased soapy flavor intensity and prevalence compared to neutral pH, and Park et al. (2014) demonstrated that WPC80 that was pre‐acidified to a pH of 3.5 had a soapy flavor that was not evident at neutral pH. There have been several studies that have noted how different variables (e.g., milk type, starter culture, and addition of enzymes) can affect the prevalence of free fatty acids (FFAs) in WPI or liquid whey (Campbell, Miracle, Drake, et al., 2011; Liaw et al., 2011; Tomaino et al., 2001). Short‐chain FFAs are naturally occurring in milk at low concentrations (K. R. Cadwallader et al., 2007), and they can increase through the addition of lipase or selection of starter culture during cheese manufacture (Campbell, Miracle, Drake, et al., 2011; Drake et al., 2009). Accordingly, FFAs are present in fluid whey and whey protein.

The source of soapy off‐flavor presented in acidified whey protein beverages has not been clearly identified. Previous studies have documented soapy flavor occasionally in whey proteins, and soapy flavor is more prevalent in acidified whey protein solutions and acidic whey protein beverages. There appears to be a relationship between pH and the presence of a soapy off‐flavor (Park et al., 2014; White et al., 2013). We hypothesize that soapy flavor is caused by medium‐chain FFAs associated with WPI that have low flavor activity under neutral pH but are more flavor active under acidic conditions. The objective of this study was to determine if FFAs, specifically octanoic, decanoic, and/or dodecanoic acids, which are known to have soapy/waxy flavors and aromas, are the cause of soapy off‐flavor in acidified WPI beverages.

## 2 MATERIALS AND METHODS

### 2.1 Samples and experimental overview

**FIGURE 1:**
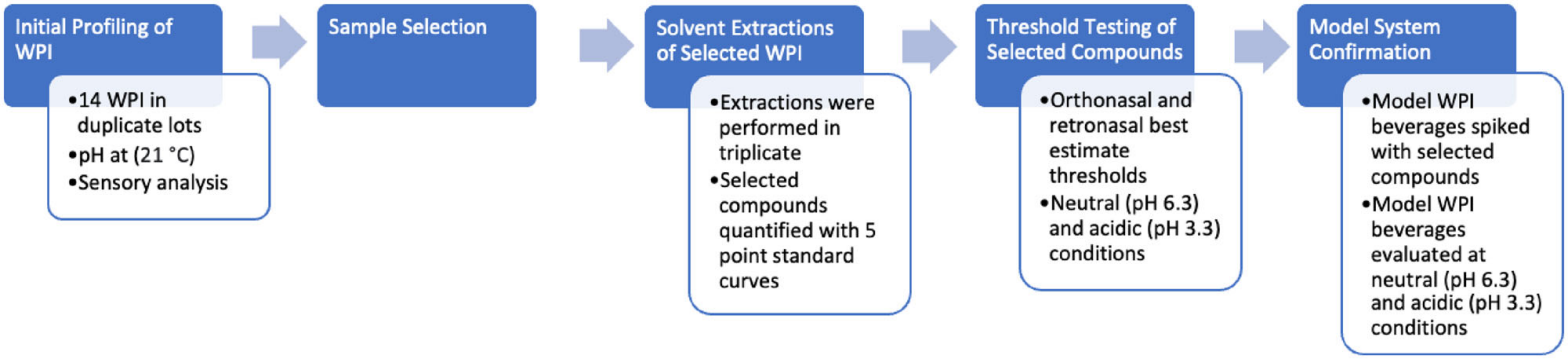
Experimental overview.

Fourteen commercial WPI powders (four pre‐acidified, five neutral pH, and five neutral pH instantized) were sourced from four different manufacturers in duplicate lots in 2021. WPI were stored at 21°C in a dark environment prior to analysis (<60 days). All sensory testing was done in accordance with North Carolina State University Institutional Review Board regulations (NCSU IRB exempted protocols 22,264 and 26,090). Each WPI was rehydrated at 10% solids (w/v) with deionized (DI) water. The pH of these rehydrated WPI was measured at 21°C. Rehydrated WPI were subjected to descriptive sensory analysis and were evaluated at their original pH, as well as pH adjusted to 6.3 (pre‐acidified) or to 3.3 (neutral) at 21°C. Neutral (pH 6.3) WPI solutions were acidified to pH 3.3 (21°C) with 0.50 M phosphoric and 0.50 M citric acids. Acidic (pH 3.3) WPI solutions were neutralized to pH 6.3 (21°C) with 2.50 M NaOH. Subsequently, six WPIs (three neutral pH and three pre‐acidified) were selected for further analyses. Selected WPIs were subjected to solvent‐assisted flavor evaporation (SAFE) with gas chromatography mass spectrometry (GCMS) and gas chromatography olfactometry (GCO) to characterize aroma‐active compounds. Quantitation of selected compounds was conducted using stable isotope dilution assays (SIDA) with deuterated standards. Retronasal and orthonasal thresholds of the selected compounds of interest were determined in both neutral (pH 6.3) and acidic (pH 3.3) conditions. Last, model WPI beverages at neutral (pH 6.3) and acidic (pH 3.3) conditions were created and spiked with the average concentrations of selected compounds quantified from SAFE. These model beverages were evaluated by a trained sensory panel (Figure 1).

### 2.2 Chemicals

Diethyl ether (anhydrous, 99.7%), methanol, hydrochloric acid (HCl, 36.5%), sodium hydroxide (NaOH, 98.7%), sodium carbonate (Na_2_CO_3_), sodium chloride (NaCl), sodium sulfate (granular, anhydrous 99%), and ethanol were purchased from Fisher Scientific. Deuterated reference standard (octanoic d‐15 acid) and other chemicals, internal standard (2‐methyl pentanoic acid), citric acid, phosphoric acid, dibasic sodium phosphate, octanoic acid, decanoic acid, and dodecanoic acid were obtained from Sigma‐Aldrich. Other deuterated reference standards (decanoic‐d19 acid and dodecanoic d‐23 acid) were purchased from CDN Isotopes. An n‐alkane series (C7–C26) was purchased from Sigma‐Aldrich.

### 2.3 pH

The pH of rehydrated WPI was determined at 21°C. WPI was rehydrated at 10% solids (w/w) in triplicate using room temperature (21°C) DI water and held overnight at 4°C to allow for complete rehydration. Fifty milliliters of rehydrated WPI was tempered to 21°C in a water bath. Subsequently, the tempered solution was placed in a 100 mL glass beaker with a stir bar on a stir plate (VMS C‐4, VWR) and stirred at a constant rate (100 rpm) while pH was taken at 21°C using a SympHony B10P pH meter (VWR) and sympHony pH probe 89,231–596 (VWR). The pH meter was calibrated at 21°C prior to sample measurement.

In order to evaluate the role of pH on flavor of WPI, neutral pH WPI was also acidified to pH 3.3 (the average pH of commercial clear acidic whey protein beverages), and pre‐acidified WPI was neutralized to pH 6.3 (the average pH of commercial neutral WPI) for sensory evaluation. Neutral pH WPI were acidified to a pH of 3.3 using 0.5 M phosphoric acid or 0.5 M citric acid at 21°C. Both of these acids were used as acidulants with the WPI, followed by sensory descriptive analysis since both are used in commercial clear acidic protein beverages. Similarly, pre‐acidified (pH 3.3) WPI were neutralized to pH of 6.3 using 2.5 M NaOH at 21°C. Prior to acidification and neutralization, preliminary experiments to determine the volumes of acid or base required to achieve the target pH for each WPI were conducted as described by White et al. (2013). The volume of 0.5 M citric acid or 0.5 M phosphoric acid to achieve a pH of 3.3 was determined, and that volume was subtracted from the total liquid needed to retain a 10% solids solution with each WPI (White et al., 2013). The same procedure was done for 2.5 M NaOH with each WPI to reach a pH of 6.3. The pH of the WPI was evaluated and confirmed using the previously described methods.

### 2.4 Descriptive analysis

WPI was rehydrated to 10% solids (w/v) in DI water as previously described. The rehydrated WPI were evaluated by a highly experienced sensory panel (*n* = 7, four females, three males, 25–53 years old), each with a minimum of 60 h previous experience in the descriptive analysis of dried dairy ingredients. An established sensory language (White et al., 2013; B. J. Wright et al., 2009) was used in conjunction with an intensity scale consistent with the Spectrum descriptive analysis method (Lawless & Heymann, 2010). The WPI solutions were dispensed (30 mL) into 64 mL lidded souffle cups with 3‐digit codes and evaluated at 21°C. WPI solutions were evaluated in duplicate by each panelist using an online ballot within the NCSU secure server network. Subsequently, six representative WPIs were selected for instrumental volatile analysis, three pre‐acidified WPI and three neutral pH WPI. These WPI were selected based on their sensory profiles; the selected neutral WPI had no or minimal soapy flavor and the pre‐acidified WPI displayed distinct soapy flavor.

### 2.5 Direct solvent extraction with solvent‐assisted flavor evaporation (DSE‐SAFE) of six selected WPIs

Volatile components of acidified, rehydrated WPI were extracted by DSE‐SAFE using a modified method from Carunchia Whetstine et al. (2005). Duplicate extractions were performed on each of the selected WPI. Acidified WPI solutions were extracted and analyzed since our focus was soapy off‐flavor in clear acidic beverages and because previous research has demonstrated that flavor active fatty acids are best extracted from matrices under acidic conditions (Carunchia Whetstine et al., 2005; Drake et al., 2010). Twenty grams of WPI was rehydrated with DI water in a 10% (w/w) solution and lowered to a pH of 1.5 using 18% HCl (v/v) (Carunchia Whetstine et al., 2005). The acidified WPI solution was evenly distributed between six (250 mL) glass centrifuge bottles (VWR). Sixty grams of NaCl (Sigma‐Aldrich) and 200 mL of diethyl ether were also added and distributed among the 6 glass centrifuge bottles (Fisher Scientific). Five milliliters of internal standard (2‐methyl pentanoic acid at 10,000 ppm in methanol) was equally distributed among the centrifuge bottles. The mixtures were shaken for 30 min at 200 rpm using an OrbiShaker (Benchmark Scientific) to separate the nonpolar solvent phase from the mixture. The bottles were then centrifuged at 1300 × g for 15 min (Thermo Lynx 6000, Thermo) to separate the ether layer. The ether layer was collected. The extraction was repeated two more times. All three extracts were combined and filtered through sodium sulfate to remove remaining water left in the ether layer. Each WPI solvent extract was then concentrated to 200 mL using a gentle stream of nitrogen and subjected to SAFE. The SAFE apparatus (Ace Glassware) was assembled and performed as described by Engel et al. (1999) and D. C. Cadwallader et al. (2022). A 1 L round flask was placed in a circulating water bath (Ace Glassware) that was set to 40°C to maintain a constant temperature. The SAFE head apparatus had two headspace traps, both of which were submerged in Nalgene buckets filled with liquid nitrogen and refilled every 20 min to maintain temperature. The entire SAFE apparatus was then brought to a vacuum of 10^−5^ torr. Once the system was equilibrated, the sample was introduced dropwise through the stopcock of the apparatus head across a 30 min span. When the sample volume had been fully introduced into the system, the system was kept at a constant vacuum and temperature for 2 h. After 2 h, the system was shut down, and the sample that was collected in the primary trap was thawed at room temperature and collected in a 250 mL amber glass jar. Phase separation procedures were followed as described by Carunchia Whetstine et al. (2005). After SAFE, the distillate was concentrated under a stream of nitrogen to 20 mL. The concentrated distillate was washed with sodium bicarbonate (0.5 M) three times and shaken vigorously for 5 min using a Multi Reax vibrating test tube shaker (Heidolph Instruments). The water phase (bottom) was removed and collected in a separate test tube. The distillate was then washed with 2 mL saturated sodium chloride solution and shaken vigorously for 5 min, three times. The water phase was removed and collected in the same tube. The solvent layer remaining was the neutral/basic fraction. This fraction was discarded since our focus was flavor active fatty acids. For the acidic fraction, the water layer was acidified to a pH of 2.0–2.5 using 18% HCl (v/v). Five milliliters of ether was added three times to the water phase and shaken vigorously for 10 min using the Multi Reax vibrating test tube shaker (Heidolph Instruments). The ether layer containing the extracted acidic volatiles was collected and was dried over anhydrous sodium sulfate. The extracted acidic volatiles were then concentrated under a stream of nitrogen to 0.25 mL.

### 2.6 GCMS analysis of solvent extracts

GCMS analysis of the solvent extracts was performed using a 7890B GC/5977B MSD (Agilent Technologies). Separations were performed using a ZB‐Waxplus (30 m length × 0.25 mm i.d. × 0.25 µm film) (Phenomenex, Torrance, CA). The method for analysis was based on Carunchia Whetstine et al. (2005) with minor modifications. Helium gas was used as a carrier at a constant flow of 1 mL/min. Oven temperature was programmed from 40°C to 200°C at a rate of 5°C/min with initial and final hold times of 5 and 45 min, respectively. Mass selective detector conditions were as follows: capillary direct interface temperature, 250°C; ionization energy, 70 eV; mass range, 33–330 amu; scan rate, 5 scans/s, with a 3 min solvent delay. Five microliters of each acidic fraction were injected in the hot splitless (250°C) mode. Triplicate injections were conducted on each extract.

### 2.7 Gas chromatography‐olfactometry

The aroma‐active components of the solvent fractions were determined by GCO. Each fraction was injected on two GCO systems (6850 GC, Agilent Technologies Inc), each with a flame ionization detector (FID) with a sniffer port (Gerstel Inc.) but different columns, ZB‐WAX plus and a ZB‐5 ms (30 m length × 0.25 mm i.d. × 0.25 µm) (Phenomenex). Five microliters of each sample were injected onto each column. The GCO method followed Whetstine et al. (2005) with minor modifications. Column effluent was split 1:1 between the FID and sniffing port. The oven was programmed to 40°C to 200°C with a rate of increase of 10°C/min with a start hold time of 3 min and a final holding time of 20 min. The FID was maintained at a temperature of 250°C. Each acidic fraction was evaluated in duplicate on both columns by two trained sniffers, each with >75 h of experience. Sniffers described the odor and rated the odor intensity on a 5‐point numerical intensity scale (Carunchia Whetstine et al., 2005).

### 2.8 Compound identification and quantitation

Compound identification was determined based on mass spectra, aroma character, retention indices on both polar and non‐polar columns, and the use of authentic standards. SIDA with deuterated standards were used to determine concentration of selected compounds. The response factors of the selected compounds were calculated through a 5‐point internal standard curve created using deuterated compounds. A stock solution of each of the deuterated standards was made in methanol. The deuterated standard stock solutions were added to rehydrated WPI that was acidified to pH 1.5 as described previously in duplicate. Each standard curve was generated by separate extractions and was injected on the same GC‐MS using the same method described previously. The selected compounds were quantified by utilizing the response factor with the area ratio of the compound to internal standards. The standard curve for octanoic acid had an *R* ^2^ 0.9925, and decanoic acid had an *R* ^2^ of 0.9929. Due to the low solubility of dodecanoic acid, the standard curve created for decanoic acid was used to quantify dodecanoic acid.

### 2.9 Threshold testing

#### 2.9.1 Orthonasal threshold testing

The orthonasal best estimate threshold (BET) of octanoic, decanoic, and dodecanoic acids was determined orthonasally in acidic (pH of 3.3) and neutral (pH 6.3) conditions. Octanoic and decanoic acids were dissolved in water‐based buffer solutions. Due to low solubility in water (<1 ppm) (American Chemical Society, 2018), dodecanoic acid was dissolved in a 40% ethanol buffer solution. To maintain a constant pH for threshold determinations, a citric acid‐dibasic sodium phosphate buffer was made (Baldwin et al., 1973). The buffer was made using 0.1 M citric acid and 0.2 M dibasic sodium phosphate solutions in DI water to generate acidic (pH 3.3) and neutral (pH 6.3) matrices. For a 3 L solution of neutral water (pH 6.3), 118 mL of 0.1 M citric acid and 182 mL of 0.2 M dibasic sodium phosphate were added to 2700 mL of DI water. For a 3 L solution of acidic water (pH 3.3), 110 mL of 0.1 M citric acid and 23 mL of 0.2 M dibasic sodium phosphate were added to 2850 mL of DI water.

All three fatty acids were dissolved in the appropriate matrix on the day of the test, and stock solutions were serially diluted by a step factor of 2. Fifteen milliliters of each signal was poured into clean, labeled 56‐mL plastic souffle cups and lidded (Solo cup). All cups were labeled with randomized 3‐digit codes. Blank samples of the corresponding matrix were added in the same amount to souffle cups and lidded. Samples were presented to consumers at 21°C. Panelists who participated in the threshold testing were instructed prior to tests (Leksrisompong et al., 2010).

Sample presentation and calculations were based on the American Society for Testing and Materials (ASTM) procedure E679–9 (ASTM, 2004). Samples were presented to panelists in a series of three with two blanks and one signal cup. Seven ascending series were tested each time. Each series was presented in a randomized order. Panelists were instructed to open the souffle cups within each series, sniff them in the corresponding presentation order, and then circle which sample they thought was different from the other two. After picking which coded sample they thought was different, panelists were then asked to indicate whether or not they were sure that the sample selected was the signal cup. The individual BETs were taken as the geometric mean of the last concentration with an incorrect response along with the first concentration that had a correct response. If the panelist indicated “not sure” on the correct choice, then a factor of 1.41 was used as an adjustment from the panelist choosing correctly by chance (Lawless et al., 2000). Group BET thresholds were taken as the geometric mean of the calculated individual BETs.

#### 2.9.2 Retronasal thresholds

Retronasal thresholds were also determined for octanoic and decanoic acids. Dodecanoic acid retronasal threshold was not performed due to the lack of solubility of dodecanoic acid and potential interference and fatigue (compound dissolved in a 40% ethanol solution). Retronasal thresholds were prepared the same way that orthonasals were prepared. Panelists were provided with a nose clip (Drake, 2007). Panelists were instructed to put on the nose clip prior to sampling of the blank and signal solutions. Panelists were instructed to place the sample in their mouth and manipulate the sample for 2–3 s. Panelists then took off the nose clip before expectorating. Calculations for retronasal BET values were calculated in the same manner as orthonasal BET values.

### 2.10 Model system confirmation

To confirm the contributions of octanoic and/or decanoic acids to soapy flavor in acidic whey protein beverages, sensory evaluation of model systems of rehydrated WPI (10% w/v) was performed at neutral (pH 6.3) and acidic (pH 3.3) conditions. The trained sensory panelists described previously evaluated the model systems. The WPIs utilized for model systems were selected from previous trained panel profiling. WPI 2 was chosen as a reference due to its soapy flavor and WPI 5 was chosen from the neutral pH WPI based on it having no soapy flavor at neutral pH and a lower intensity of soapiness compared to the other neutral pH WPI when acidified with phosphoric acid (Table 1). Neutral models were determined using neutral pH WPI 5 (pH 6.3) and by neutralizing pre‐acidified WPI 2 to pH 6.3 with 2.5 M NaOH. Acidic models were performed using pre‐acidified WPI 2 (pH 3.3) and by acidifying a neutral pH WPI 5 to an acidic pH with 0.5 M citric or 0.5 M phosphoric acids. Model systems were not performed on dodecanoic acid due to its low solubility in water.

**TABLE 1:** Trained panel soapy flavor intensity of selected rehydrated (10% w/v) whey protein isolate (WPI) at acidic and neutral pH at 21°C.

	Soapy flavor intensity (0–15 pts)			
WPI	pH 6.3	pH 3.3 (commercial pre‐acidification)	pH 3.3 (phosphoric acid)	pH 3.3 (citric acid)
1	ND	1.6a	–	–
2	ND	1.2b	–	–
3^*^	ND	1.5a	–	–
4	ND	–	1.5b	1.3b
5	ND	–	1.0c	1.3b
6^*^	1.0	–	2.6a	1.6a

*Note*: Samples were evaluated by a trained panel (*n* = 7) in duplicate using a 0–15 point intensity scale. Samples 1–3 are commercial pre‐acidified WPI and Samples 4–6 are commercial neutral pH WPI. Different letters in columns following means signify significant differences (*p* < 0.05). If the same letters occur, then there is no significant difference between those means (*p* > 0.05). WPI were evaluated as‐is and then acidified to pH 3.3 (neutral pH WPI) or neutralized to pH 6.3 (commercial pre‐acidified WPI).

Abbreviation: ND, not detected.

* Ion exchange WPI.

The average concentration of octanoic and decanoic acids was used to spike acidified WPI (pH 3.3) and neutral WPI (pH 6.3) solutions. Stock solutions of each acid were made and added to rehydrated WPI. Octanoic and decanoic acids were evaluated separately and in combination. The following references were provided: a rehydrated, pre‐acidified WPI that was previously determined by the trained panel to have a soapy aroma and flavor (soapy reference) and a rehydrated neutral WPI powder that had no soapy characteristics and blank (unspiked) solutions of the acidic and neutral WPI that were selected for model system matrices. The baseline sensory profile of the acidified and neutral WPI was confirmed by the trained panel prior to the addition of the compounds. Each compound or compound combination in each WPI model along with a coded unspiked solution (blind control) was dispensed into lidded soufflé cups with three‐digit codes and was then evaluated for soapy flavor intensity using the 0–15 point intensity scale described previously. Each coded sample was also scored on a similarity scale of 0–10, with 0 being not similar at all and 10 being highly similar compared to the pre‐acidified soapy WPI (soapy reference). Each WPI model was evaluated in duplicate by each panelist at room temperature (21°C).

### 2.11 Statistical analyses

Analysis of variance with Tukey's post hoc test was performed on trained panel and compound quantitation data. Statistical analyses were performed with XLSTAT (2022, Addinsoft Inc.) and carried out at 5% significance level.

## 3 RESULTS AND DISCUSSION

### 3.1 Commercial WPI

The average pH of the 4 pre‐acidified WPI at 21°C was 3.31 ± 0.12, and the average pH of the 10 neutral (or regular) WPI at 21°C was 6.28 ± 0.43. These reported values and the pH range are similar to other studies that examined the pH of commercial WPI under acidic and neutral conditions (Park et al., 2014; White et al., 2013). Neutral ion‐exchange WPI 6 had a significantly higher (*p* < 0.05) pH than the other neutral WPI (7.07 ± 0.12 vs. 6.28 ± 0.43) as expected since the process of manufacture of this WPI is distinct.

### 3.2 Sensory profiles of rehydrated WPI

Sensory profiles of the 10 neutral rehydrated WPIs were generally similar with variable intensities of cardboard and sweet aromatic flavors and astringency, typical of rehydrated WPI (results not shown) (B. J. Wright et al., 2009). Pre‐acidified WPI were characterized by cardboard, sour aromatic, and soapy flavors, sour taste, and astringency (results not shown), also consistent with previous work (Park et al., 2014). Six representative WPI (three neutral and three pre‐acidified) were selected for further analyses. Pre‐acidified WPI were selected based on high soapy flavor intensity. Neutral pH counterparts from the same manufacturer were also selected. The pre‐acidified WPI had low but distinct soapy flavor (mean 1.2–1.6 on a 0–15 point intensity scale) when rehydrated (pH 3.3). However, when WPI 1–3 were neutralized to pH 6.3, soapy flavor was not detected (Table 1). Neutral WPI 4–6 (pH 6.3) had no soapy flavor except for WPI 6 (Table 1). WPI 6 had a pH of 7.07 ± 0.12 after rehydration, higher (*p* < 0.05) than the other neutral pH WPI (6.28 ± 0.43), which may explain the perceived soapy flavor. Additionally, WPI 6 was an anion exchange WPI, and the soapy flavor (and higher pH) may also be due to the distinct manufacturing process, which involves a salt or mild pH adjustment rather than traditional microfiltration (Carter & Drake, 2018). When the neutral pH WPI was acidified with phosphoric or citric acid, a soapy flavor was evident, and the soapy flavor increased in WPI 6 (Table 1).

Previously, Park et al. (2014) reported that when liquid WPC80 retentate was acidified to pH 3.5 with a blend of phosphoric and citric acids prior to spray drying, soapy flavor was evident in the reconstituted pH 3.5 powder and was not detected in the same liquid WPC80 retentate acidified to pH 5.5 or 6.5 prior to spray drying. Concurrently, the WPC80 powders that were spray dried at pH 5.5 or 6.5 had a soapy flavor when the reconstituted powder was acidified to pH 3.5 prior to sensory evaluation. Park et al. (2014) noted that the process of pre‐acidification reduced neutral volatile aldehydes and accordingly, reduced cardboard flavor in the spray dried (pH 3.5) powder. They hypothesized that the process of pH reduction increased partitioning of volatile neutral aldehydes into the headspace and allowed more loss of these compounds during spray drying. Park et al. (2014) also noted that the source of soapy flavor was unknown. Soapy flavor has been previously documented in WPC80 and WPI that were not under acidic conditions (Drake et al., 2009; Evans et al., 2010) and was also documented in WPI 6 in the current study. Acidic conditions (namely the acidulants) are not the source of the soapy flavor. The acidulants in the current study were evaluated at pH 3.3 prior to use with WPI, and neither were determined to have a soapy flavor. Soapy flavor was documented in pre‐acidified WPI, but also when neutral pH WPI were acidified with different acidulants (phosphoric and citric acid) to pH 3.3. Only neutral WPI 6 had a low intensity of soapy flavor at a neutral pH, and acidification increased soapy flavor intensity (Table 1). Decrease in pH increased the prevalence and intensity of soapy flavor in the current study and in other studies (Park et al., 2014; White et al., 2013).

### 3.3 Identification and quantitation of octanoic, decanoic, and dodecanoic acids

Medium‐chain FFAs have been documented previously in whey proteins (Carunchia Whetstine et al., 2005; Evans et al., 2010; Mortenson et al., 2008; White et al., 2013). These acids have distinct soapy flavors (Carunchia Whetstine et al., 2005; McDaniel et al., 1969) and have been used as trained panel references for soapy flavor (Cadwallader et al., 2007; Whetstine et al., 2005). These three acids were the focus of the current study. Concentrations of octanoic, decanoic, and dodecanoic acids were within a similar range in both pre‐acidified and neutral WPIs (Table 2). All three acids were also aroma‐active by GCO of solvent extracts in the six selected WPIs and each acid was described as soapy in aroma at the sniffer port.

**TABLE 2:** Mean concentrations of selected volatile free fatty acids in whey protein isolate (WPI).

	WPI	Concentration of acid (ppm) mg/kg		
		Octanoic	Decanoic	Dodecanoic
Pre‐acidified WPI	1	16.5a ± 0.3	28.8ab ± 1.6	25.9a ± 0.8
	2	13.7bc ± 1.2	27.4bc ± 1.6	26.5a ± 2.6
	3^*^	13.0c ± 0.6	26.4c ± 2.1	23.7b ± 0.7
Neutral WPI	4	16.5a ± 2.1	30.2a ± 0.9	22.7b ± 0.7
	5	13.6bc ± 2.6	26.7bc ± 3.5	25.83a ± 3.4
	6^*^	14.9ab ± 1.7	28.4abc ± 1.2	23.8b ± 0.8

*Note*: Samples were evaluated by triplicate extractions and injections. Different letters in columns following means signify significant differences (*p* < 0.05). If same letters occur, then there is no significant difference between those samples (*p* > 0.05).

* Ion exchange WPI.

Carunchia Whetstine et al. (2005) reported that WPI that had a higher relative abundance of octanoic and decanoic acids had a higher intensity of soapy flavor. Their results suggested that these two acids contributed to the soapy flavor of WPI (Carunchia Whetstine et al., 2005). However, Carunchia Whetstine et al. (2005) did not evaluate pre‐acidified WPI nor did they quantify these medium chain fatty acids. They also did not report acids higher than C6 as aroma active. Carunchia Whetstine et al. (2005) reported the relative abundance of octanoic and decanoic acids in WPI from 0.89 to 39.00 ppm and 9.50 to 157.00 ppm, respectively. Table 2 shows the concentration of octanoic acid ranging from 13.0 to 16.5 ppm and decanoic acid ranging from 26.4 to 30.2 ppm across the six WPIs evaluated in this study.

Short‐chain FFAs are present naturally in milk fat at very low concentrations (K. R. Cadwallader et al., 2007) and are also present in fluid whey (Karagül‐Yüceer et al., 2006; Tomaino et al., 2001). Campbell, Miracle, Gerard, et al. (2011) reported that skim milk and whey from skim milk had higher concentrations of FFA than whole milk and whey from whole milk; it has also been reported that skim milk has a higher amount of phospholipids than whole milk (Campbell, Miracle, Gerard, et al., 2011; Morr & Ha, 1993). Carunchia Whetstine et al. (2003) confirmed that FFA concentration can be affected by a number of factors, including milk source (percent fat, cow breed, and feed type) and starter cultures (mesophilic vs. thermophilic), along with processing and handling procedures (Campbell, Miracle, Drake, et al., 2011; Carunchia Whetstine et al., 2003). Drake et al. (2009); Croissant et al., 2007 confirmed that the starter cultures used for cheesemaking (thermophilic vs. mesophilic) changed the sensory perception and volatile compound make‐up of the liquid whey. Tomaino et al. (2001) showed distinct differences in liquid whey depending on what starter cultures were used; liquid whey made with *Lactococcus lactic* subsp. *Lactis* had the highest concentration of FFAs among the starter cultures evaluated in their study (Tomaino et al., 2001). Liaw et al. (2011) studied flavor differences of whey from Cheddar and Mozzarella production and found that Cheddar whey had the highest level of lipid oxidation. While levels of lipid oxidation do not directly relate to FFA concentration, these studies show how starter culture and cheese type can affect the compositional make up of liquid whey (the source of WPI). FFA content in dairy products can also increase with the addition of lipases, which may be used in Italian and Feta cheese manufacture (Drake et al., 2009). Lipases can increase the concentration of FFAs present in the liquid whey along with imparting undesirable flavors (Drake et al., 2009).

### 3.4 Threshold determination of octanoic, decanoic, and dodecanoic acids

Orthonasal BET values for octanoic and decanoic acids in water‐based buffer solutions was lower than previously reported at similar pH ranges (Baldwin et al., 1973; Table 3). Baldwin et al. (1973) did not lid their samples after pouring, nor did they use an established threshold procedure such as the ASTM procedure E679–9 (ASTM, 2004) utilized in the current study. Orthonasal thresholds of dodecanoic acid were higher than thresholds of decanoic or octanoic acids (Table 3). This result was expected, because dodecanoic acid has a higher molecular weight than octanoic and decanoic acids, making it less volatile than shorter chained fatty acids. Dodecanoic acid also has lower solubility in water and was dissolved in 40% ethanol for orthonasal threshold testing. Retronasal threshold of dodecanoic acid was not determined due to the interference of alcohol burn that was caused by such a high alcohol content. Retronasal thresholds of octanoic and decanoic acids were higher than orthonasal thresholds (Table 3). Other studies with thresholds have also reported some compounds having a higher retronasal threshold than orthonasal BET values (Piornos et al., 2019). These results align with the theory of the duality of the olfactory sense, which essentially states that the route of detection changes the perception of the odor (Rozin, 1982). As expected, the BET values of the three acids were lower under acidic conditions than at neutral pH (Table 3). FFAs are more easily detectable at a lower pH because the anions are protonated in an acidified pH, which increases volatility of FFAs by decreasing polarity (Bills et al., 1969). The concentration of these acids in WPI (Table 2) fall within a similar range regardless of initial WPI pH. Taking into account sensory thresholds, these acids could be flavor active at low pH and not detected at neutral pH.

**TABLE 3:** Orthonasal and retronasal best estimate thresholds (BET) (mg/kg) results at 21°C.

Compound	Orthonasal BET		Retronasal BET	
	Acidic (pH 3.3) (mg/kg)	Neutral (pH 6.3) (mg/kg)	Acidic (pH 3.3) (mg/kg)	Neutral (pH 6.3) (mg/kg)
Octanoic acid	0.422b ± 0.32	2.34a ± 0.42	0.904b ± 0.41	3.91a ± 0.12
Decanoic acid	0.117b ± 0.38	2.82a ± 0.41	0.415b ± 0.55	3.05a ± 0.23
Dodecanoic acid	28.9b ± 0.41	141a ± 0.36	–	–

*Note*: Different letters in rows following means signify significant differences (*p* < 0.05). Dodecanoic acid retronasal thresholds were not performed due to low solubility of C12 in water. Sample size for orthonasal results: Octanoic acid (*n* = 45); decanoic acid (*n* = 60); dodecanoic acid (*n* = 40). Sample size for retronasal results: Octanoic acid (*n* = 45); decanoic acid (*n* = 35).

### 3.5 Model system confirmation

**FIGURE 2:**
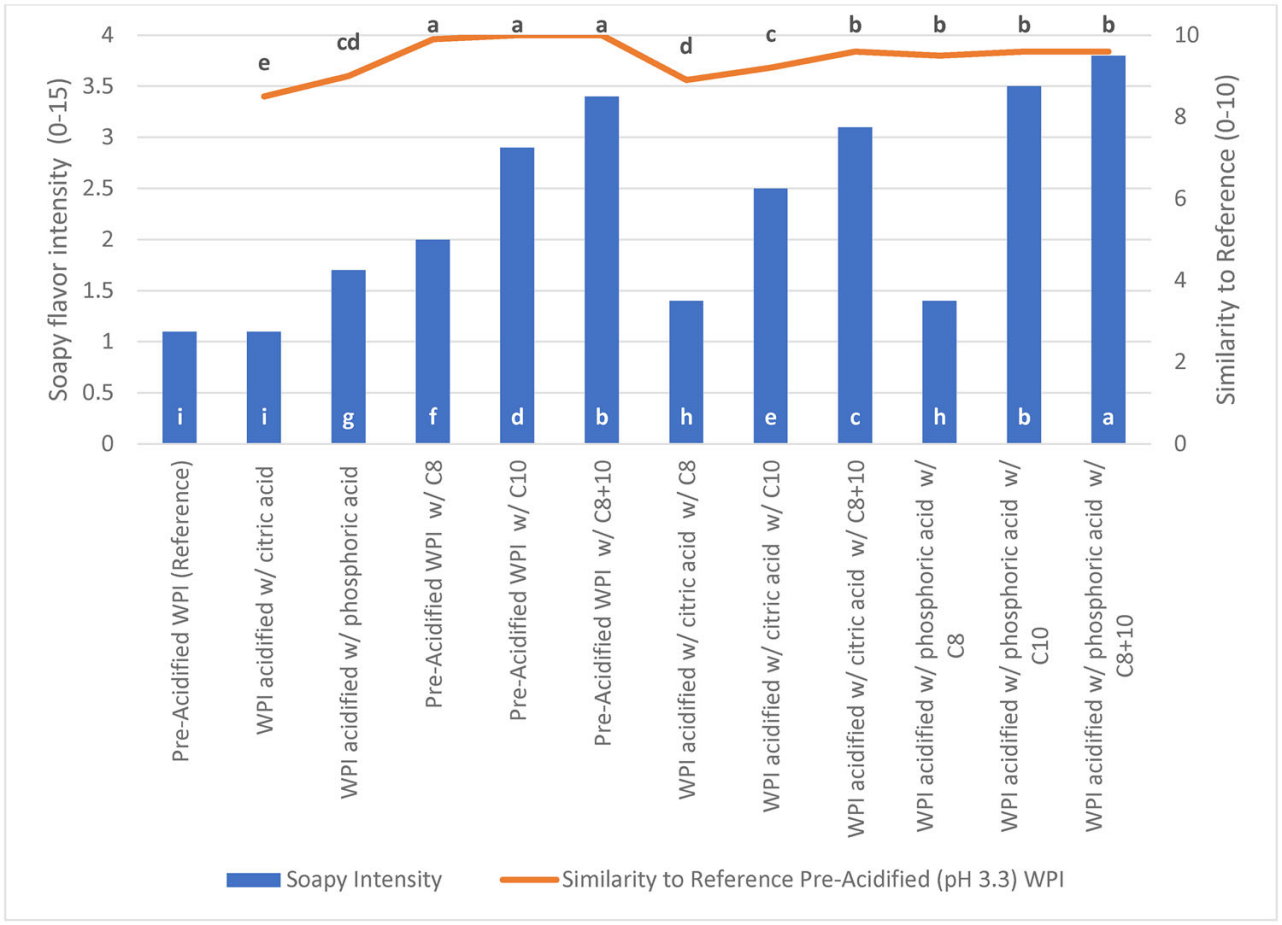
Trained panel acidic model system results (pH 3.3). Samples were evaluated by a trained panel (*n* = 7) in duplicate using a 0–15 point intensity scale at 21°C. Each coded sample was also scored on a similarity scale of 0–10, with 0 being not similar at all and 10 being highly similar compared to the commercial pre‐acidified soapy whey protein isolate (WPI) (soapy reference). Different letters signify differences (*p* < 0.05). Amounts of each acid added to WPI solution for each combination: C8 = 14.4 mg/kg; C10 = 27.8 mg/kg. Sample labeled “pre‐acidified” was received as a commercial pre‐acidified WPI powder.

Whey protein model systems containing added concentrations of the targeted FFAs (octanoic and decanoic) were prepared to further confirm specific contributions to soapy flavor in clear acidic whey protein beverages. The model systems were evaluated by a trained sensory panel at neutral (pH 6.3) and acidic (pH 3.3) conditions with and without the addition of each fatty acid alone and in combination. The fatty acids were added at the average concentrations found in WPI. Commercial pre‐acidified WPI was used as a control and point of reference for the trained panel. Addition of C8 or C10 to pre‐acidified WPI solution increased soapy flavor intensity, while neutralization of these same solutions to pH 6.3 resulted in no detectable soapy flavor (results not shown). Neutral pH WPIs acidified with either citric or phosphoric acid had a low intensity of soapy flavor, presumably due to native FFAs present in the WPI. When C8 or C10 were added to neutral pH WPI and acidified, soapy flavor intensity increased compared to WPI that was acidified only (Figure 2). The panel described octanoic acid as having an earthy/soapy flavor, whereas decanoic acid had an ivory soap bar flavor and aroma. Dodecanoic acid was not tested in model systems due to its low solubility, but its aroma was described as waxy/soapy. Trained panelists noted that decanoic acid alone was the closest sensory match in soapy character to the pre‐acidified reference WPI. Addition of these acids alone or in combination to neutral pH WPI elicited no detectable soapy flavor (results not shown). These WPI model systems confirm the role of octanoic and decanoic acids as contributors to soapy flavor in acidified whey protein beverages.

Soapy flavor was evident in acidified WPI, regardless of the acidulant used (Figure 2). WPI acidified with citric acid to pH 3.3 that contained C8 + C10 matched closer to the pre‐acidified soapy WPI reference than when the acids were added individually to WPI acidified with citric acid (*p* < 0.05). In contrast, all combinations of fatty acids added to WPI acidified with phosphoric acid were similar to the reference pre‐acidified WPI (*p* > 0.05). Soapy intensity was the highest for the WPI that was acidified (pH 3.3) with phosphoric acid and spiked with C8 + C10 (*p* < 0.05). Pre‐acidified WPI (pH 3.3) spiked with C8 + C10 had the second highest soapy intensity (*p* < 0.05), and WPI acidified with citric acid (pH 3.3) spiked with C8 + C10 was the third most intense soapy flavor (*p* < 0.05).

Previous studies have shown that pH plays a large role in the flavor profile of dairy foods. Woo et al. (1984) analyzed the FFA concentration of various cheeses. Limburger cheese was found to have around the same amount of FFA as other cheeses evaluated; however, the flavor of the Limburger cheese did not indicate a presence of FFA (e.g., rancid flavor); this was likely due to the pH of this cheese (pH 6.97) being much higher than the other cheeses (Woo et al., 1984). The higher pH results in a greater amount of the ionized form of the fatty acids, which makes them less flavor active (Woo et al., 1984). Under acidic conditions (pH 1–1.5), FFA aromas were much more abundant, presumably due to the acids being primarily in protonated form (Woo et al., 1984). White et al. (2013) reported that WPIs that were heated and acidified (pH 3.2) had higher soapy flavor than a neutral pH WPI. Evans et al. (2010) manufactured acidic protein beverages using WPC80 and reported low intensities of soapy flavor; however, when the same WPC80 was rehydrated and adjusted to a neutral pH, there was no soapy flavor documented (Evans et al., 2010). These studies show a direct relationship between soapy flavor and pH in whey protein that was also evident with FFAs and pH in cheeses (Woo et al., 1984). The current study demonstrates a similar relationship between medium‐chain FFAs, namely, octanoic and decanoic acids, in WPI and pH. These acids are present at variable concentrations in spray dried whey proteins. The pKa value of octanoic acid is 4.9, and 6.4 for decanoic acid. At a pH of 3.3, >95% of each of these acids would be protonated. In contrast, at pH 6.3, >95% of octanoic acid and approximately 44% of decanoic acid would exist in the ionized form. The ionized form of these acids would be more polar and less volatile in an aqueous system compared to the predominant protonated form at pH 3.3. At their concentrations under neutral pH, they are primarily ionized and below sensory detection. However, under conditions of clear acidic protein beverages (pH 3.3), these fatty acids are largely in protonated form, and concentrations may be sufficient to be at flavor active concentrations (e.g., concentrations > sensory BET values). More in‐depth studies relating to modifications of whey protein processing could be done to identify possible processing changes to minimize soapy off‐flavor in pre‐acidified WPI. One key component of whey processing that can be evaluated is the starter cultures used for cheesemaking and/or addition of lipases. When choosing the type of whey to use to produce pre‐acidified WPI, manufacturers should choose whey that is known to have a lower fatty acid profile. These results can specifically aid in the production of pre‐acidified WPI for appealing clear acidic protein beverages.

## 4 CONCLUSION

Soapy flavor was present in WPI under acidified conditions (pH 3.3) with and without addition of octanoic and decanoic acids and regardless of acidulant used. Results from this study suggest that the soapy flavor in acidified whey protein beverages is caused by a mixture of low concentrations of octanoic and decanoic acids associated with the (spray dried) whey protein. While dodecanoic acid has a soapy aroma and flavor, it is likely not the cause of this off‐flavor due to its aroma being different than what was detected in WPI and due to its low solubility in water. Orthonasal and retronasal threshold results along with sensory analysis of model systems show that these acids are not flavor active at neutral pH, but when the pH of a whey protein solution is decreased to pH 3.3, a soapy flavor is evident.

## AUTHOR CONTRIBUTIONS

**Anita R. Best**: Investigation; writing—original draft; formal analysis; data curation; writing—review and editing. **Yaozheng Liu**: Methodology; formal analysis; data curation. **Dylan Cadwallader**: Methodology; formal analysis; data curation. **MaryAnne Drake**: Conceptualization; investigation; funding acquisition; writing—review and editing; project administration; supervision.

## CONFLICT OF INTEREST STATEMENT

The authors declare no conflicts of interest.
